# The clinical effect of a strategy called transcystic gallbladder-preserving cholecystolithotomy based on endoscopic retrograde cholangiopancreatography for cholecystolithiasis: A retrospective study from a single center

**DOI:** 10.3389/fsurg.2022.1021395

**Published:** 2023-01-06

**Authors:** Zhenzhen Yang, Junbo Hong, Liang Zhu, Cheng Zhang, Xiaojiang Zhou, Guohua Li, Yong Zhu, Zhijian Liu, Xiaodong Zhou, Youxiang Chen

**Affiliations:** ^1^ Department of Gastroenterology, The First Affiliated Hospital of Nanchang University, Nanchang, China; ^2^ Department of Ultrasound, The First Affiliated Hospital of Nanchang University, Nanchang, China

**Keywords:** choledocholithiasis combined with cholecystolithiasis, endoscopic retrograde cholangiopancreatography, gallbladder function, gallbladder-preserving cholecystolithotomy, retrospective study

## Abstract

**Background:**

Choledocholithiasis complicated with cholecystolithiasis is a common disease. This study explores a novel strategy, called ERCP-based transcystic gallbladder-preserving cholecystolithotomy, for the simultaneous removal of common bile duct stones and gallbladder stones.

**Methods:**

From December 2018 to June 2021, all patients with cholecystolithiasis and common bile duct stones who met the criteria for gallbladder preservation in our hospital were included in the study and prospectively followed up.

**Results:**

We included 48 patients, including 20 patients with acute biliary pancreatitis. All patients successfully underwent ERCP to remove common bile duct stones. One patient had gallbladder perforation during gallbladder-preserving cholecystolithotomy. The guide wire successfully entered the gallbladder, and the transpapillary gallbladder metal-covered stent was successfully placed in 44 patients. The technical success rate was 91.67% (44/48). All stones were removed in 34 patients, for a clinical success rate of 77.27% (34/44). The total postoperative complication rate was 6.25% (3/48), with 2 cases of pancreatitis (4.17%) and 1 case of cholangitis (2.08%). Three patients were lost to follow-up. Among the 31 patients who were followed up for a mean of 27 months (6–40), 5 patients (16.13%) experienced gallstone recurrence. The recurrence rates at 12 months, 18 months, 24 months, 30 months and 36 months were 0%, 3.23%, 6.45%, 12.9%, and 16.13%, respectively.

**Conclusion:**

For patients with cholecystolithiasis and common bile duct stones, ERCP-based transcystic gallbladder-preserving cholecystolithotomy without gallbladder incision can preserve gallbladder structure, and this procedure is safe and feasible for the protection of gallbladder function.

**Clinical trial registration:** The study was registered in the Chinese Clinical Trial Registry, and the registry number is ChiCTR1900028006.

## Introduction

Benign biliary diseases, represented by common bile duct stones and gallbladder stones, are common diseases in the world today. With the improvement in people's living standards and the increase in societal ageing, the incidence of these diseases is on the rise. The total prevalence of gallbladder stones in adults worldwide has reached 20% (1). In China, approximately 10%–15% of patients with gallbladder stones have common bile duct stones (2). Guidelines (2) recommend that common bile duct stones should be treated regardless of symptoms, and endoscopic retrograde cholangiopancreatography (ERCP) is the main treatment method. Clinically, because of the concern that gallstones will enter the common bile duct and cause common bile duct stones again or even induce biliary pancreatitis, most patients hope that gallstones can be treated at the same time as common bile duct stones. For many years, laparoscopic cholecystectomy has been the main treatment for gallstones. However, with the recognition of the importance of gallbladder function and the side effects after cholecystectomy, an increasing number of scholars advocate gallbladder preservation (3–6). To preserve the integrity of the gallbladder structure and protect its function while removing stones, we devised a new minimally invasive treatment technique for gallbladder preservation inspired by endoscopic nasocystic duct drainage and transpapillary gallbladder stents in the treatment of acute cholecystitis (7–9). Since December 2018, our new approach, called ERCP-based transcystic gallbladder-preserving cholecystolithotomy, has been performed for 48 patients. This report details our study observations.

## Method

### Selection

According to our previous experience and published literature (10), patients who met the following criteria were included in our study: preoperative abdominal B-ultrasound and MRCP and other imaging examination findings clearly indicated a diagnosis of concomitant gallbladder and common bile duct stones, and B-ultrasound showed that the morphology and size of gallbladder was essentially normal; the patient had a strong desire to preserve the gallbladder; the thickness of the gallbladder wall was ≤3 mm; gallbladder stones were less than 1 cm in diameter;and the size of common bile duct stones was ≤1.2 cm. Exclusion criteria: a history of ERCP treatment; a history of previous biliary tract surgery; concomitant gallbladder and common bile duct stones associated with atrophic cholecystitis; porcelain gallbladder; suspected malignant tumour of the gallbladder; stenosis of the lower segment of the common bile duct; Mirrizzi syndrome; coagulation dysfunction and bleeding diseases; and other contraindications for ERCP. After screening and evaluation, a total of 48 patients were enrolled between December 2018 and June 2021 (Table 1). All procedural protocols were approved and supervised by the ethics committee of our hospital.

**Table 1:** Flow diagram of the patient selection process.

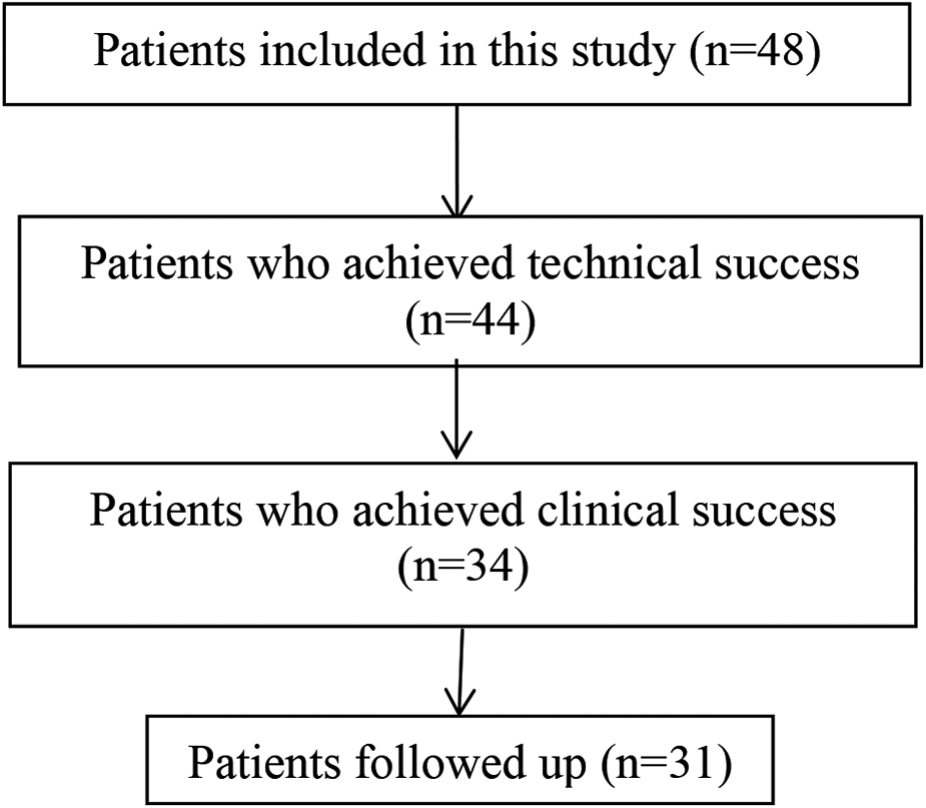

### Patients

The 48 patients included in the study included 23 males and 25 females, with an average age of 46.3 center14.6 years (range, 21–77 years). Twenty-eight patients were diagnosed with common bile duct stones and gallbladder stones (Group A), and 20 patients were diagnosed with acute pancreatitis, common bile duct stones and gallbladder stones (Group B). The average course of disease was 53.7 days, the shortest was 1 day, and the longest was 2 years. All the patients had abdominal pain symptoms of varying degrees, 3 patients had fever, 2 patients had nausea and vomiting, and 19 patients had yellow staining of the skin and sclera. In total, 18 patients had concomitant diseases, including hypertension in 7 patients, diabetes in 5 patients, chronic hepatitis in 2 patients, emphysema in 2 patients, and 1 patient each with hyperlipidaemia and chronic kidney disease (Table 2).

**Table 2:** Characteristics of patients.

	Value
Patients (*N*)	48
Male (*n*)	23
Female (*n*)	25
Age range (year)	21–77
Average age	46.3 ± 14.6
No preoperative pancreatitis (Group A) (*n*)	28
Preoperative complicated acute pancreatitis (Group B) (*n*)	20
With acute biliary pancreatitis, mild	15
With acute biliary pancreatitis, moderately severe	4
With acute hypertriglyceridaemia + biliary pancreatitis, mild	1
With hypertension (*n*)	7
With diabetes (*n*)	5
With chronic hepatitis (*n*)	2
With pulmonary emphysema (*n*)	2
With chronic nephrosis (*n*)	1
With hyperlipidaemia (*n*)	1
History of surgery (*n*)	9
History of cholecystolithiasis ≥10 years (*n*)	3

Stone conditions: (1) gallbladder stones: ① 3 patients had sediment-like stones; ② 45 patients had non-silt-like stones, including 12 patients with single stones and 33 patients with multiple stones (3 patients with more than 10 stones), the smallest was 0.4 cm, and the largest was 2 cm, with an average of 0.9 cm; (2) common bile duct stones: ① 24 patients had sediment-like stones, among whom 16 patients were diagnosed with acute biliary pancreatitis; ② 24 patients had non-silt-like stones, and the average size was 0.7 cm (0.4 cm–1.2 cm).

### Equipment

The instruments used in this study included a duodenoscope (Olympus TJF-240/TJF-260V; Olympus, Tokyo, Japan), a SpyGlass peroral cholangioscopy (Boston Scientific, Boston, America), several guidewires (0.035 inch; Boston Scientific, America), a catheter (PR-104Q-1; Olympus, Tokyo, Japan or OE-104-2225DL; EndoFlex, Voerde, Germany), a full-covering metal stent (10 cm/12 cm × 1.0 cm; Nanjing MicroPort, Nanjing, China), a biliary dilation balloon(5.5 cm × 1.0 cm; Nanjing MicroPort, Nanjing, China), and so on.

### Operative procedures

**Figure 1:**
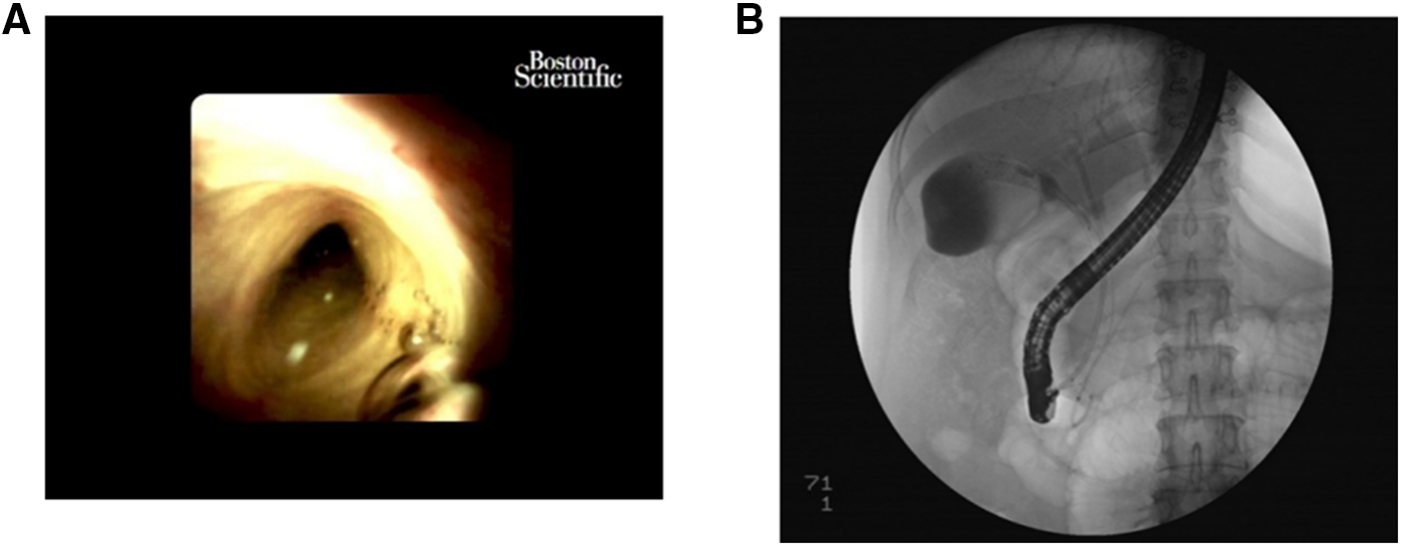
The first stage. (**A**) The opening of the cystic duct could easily be identified by SpyGlass peroral cholangioscopy. (**B**) The FCMS was introduced into the cystic duct.

For the patients in this study, we performed this operation in two stages. In the first stage, ERCP was performed under general anaesthesia. Through the major duodenal papilla, cannulation into the common bile duct was the key process before contrast injection, and a pancreatic duct stent was indwelled if the guide wire entered the pancreatic duct during intubation. Then, mini-incision endoscopic sphincterotomy (MI-EST) and/or endoscopic papillary small balloon dilation (EPSBD) was performed, and the common bile duct stones were extracted. By fluoroscopic guidance or SpyGlass peroral cholangioscopy (Figure 1A), the opening of the cystic duct could easily be identified, and then a single guidewire was introduced into the gallbladder through the cystic duct under fluoroscopic guidance followed by repeated adjustment of the guidewire direction. The other wire penetrated into the common bile duct with a plastic stent implantation that went beyond the cystic duct to support the patency of the biliary tract. The full-covering metal stent (FCMS) was introduced into the cystic duct (Figure 1B). If necessary, 7 Fr, 8.5 Fr, and 9 Fr dilatation catheters were used to dilate the cystic duct step by step prior to FCMS introduction. The proximal end of the stent crossed the gallbladder neck and opened into the gallbladder cavity, and the distal end opened into the large duodenal papilla or the lower segment of the common bile duct in the papilla. Finally, nasobiliary drainage was performed with irrigation by injecting normal saline against the FCMS tunnel, especially for muddy stones in the gallbladder.

**Figure 2:**
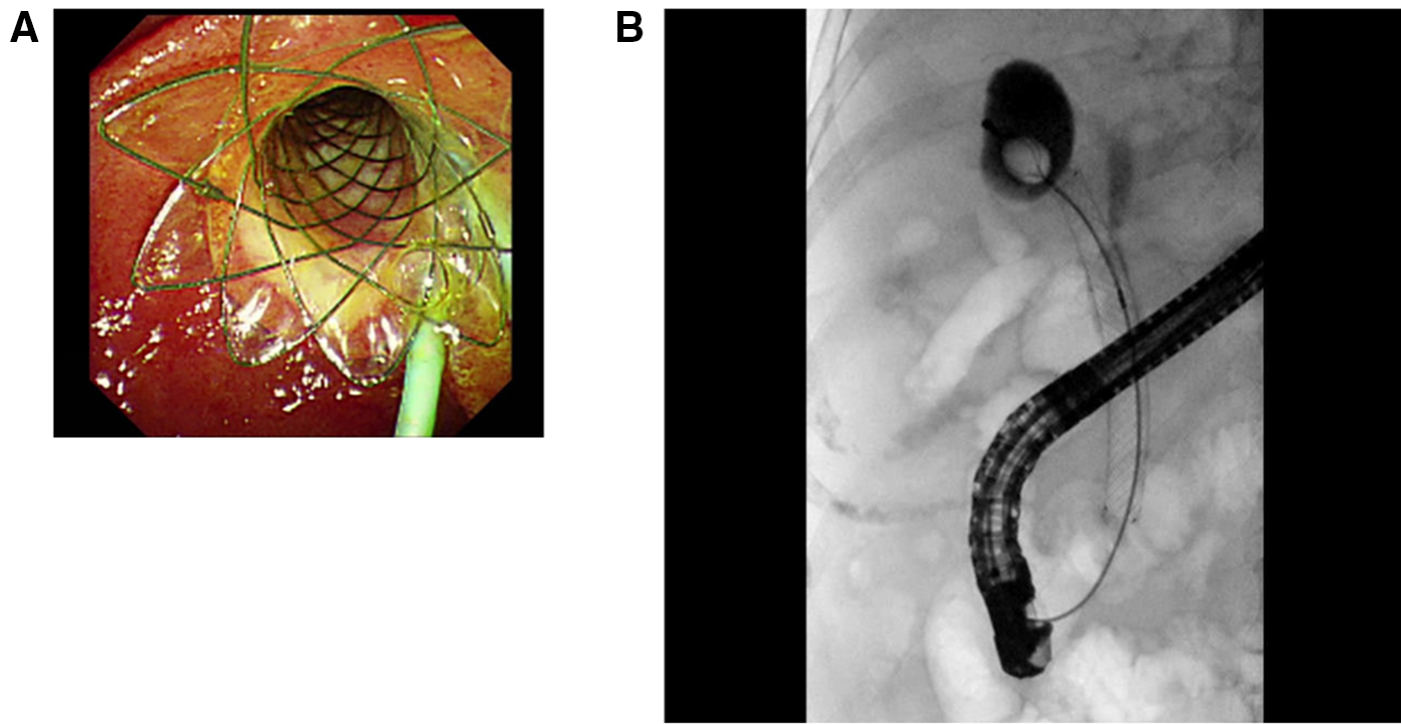
The second stage. (**A**) It could be seen that the stent expanded well 48 h after FCMS placement. (**B**) Gallbladder stones were removed by a stone basket through the channel from the duodenal papilla to the gallbladder established by transcystic FCMS.

In the second ERCP stage, therapeutic ERCP was conducted approximately 48 h or 72 h after FCMS placement for full stent expansion (Figure 2A), making it easier to perform gallstone extraction. First, nasocystic duct angiography was implemented to demonstrate the situation in the gallbladder. If stone extraction was needed, the drainage tube of the nasal gallbladder was removed. Then, through the channel from the duodenal papillary to gallbladder established by transcystic FCMS, gallbladder stones were removed by a stone basket (Figure 2B); if necessary, a gravel basket could be used. Finally, after the clearance of the gallstones, both the plastic biliary stent and the FCMS were pulled out with grasping forceps.

### Postoperative treatment

According to routine ERCP postoperative care, patients were fasted and rehydrated on the day of the operation, antibiotics were continued for patients with biliary tract infections before the operation, and antibiotics were used preventively for patients without biliary tract infections before the operation. After the first ERCP, the serum amylase level was monitored 3 h and 24 h after the operation to determine the patient's vital signs and abdominal symptoms and signs. For patients without preoperative symptoms related to biliary tract infection, such as fever, if chills and fever occur after surgery, it is considered postoperative cholangitis, and anti-infective treatment should be strengthened. For patients without preoperative acute biliary pancreatitis, if postoperative 3-hour and 24-hour blood amylase was higher or even more than 3 times than the normal value, but there was no abdominal pain consistent with pancreatitis, the patient was considered to have hyperamylasaemia, but no additional special treatment was needed. If 2 of the following 3 characteristics were present, postoperative pancreatitis was diagnosed (11): (1) abdominal pain was consistent with acute pancreatitis, (2) serum amylase and/or lipase concentrations were at least 3 times higher than the upper limit of normal, and (3) the abdominal imaging examination was consistent with the imaging changes of acute pancreatitis. Patients with postoperative pancreatitis were treated with pancreatic enzyme secretion inhibitors, pancreatic enzyme activity inhibitors, acid suppressors and stomach protectants, along with treatment to promote rehydration and defecation.

### Follow-up

Every patient for whom gallbladder stone extraction was successful (the technique was successful, and all the stones in the gallbladder were removed) was followed up after surgery, and abdominal B-ultrasound was performed every 6 months until May 1, 2022. B ultrasound was used to understand the shape and size of the patient's gallbladder and to determine whether there was a recurrence of stones or whether there were other lesions of the gallbladder, such as gallbladder polyps. The patient's improvement in symptoms was recorded.

### Statistical analysis

Statistical analysis was performed using SPSS 17.0 statistical software. The continuous variables are presented as the mean with standard deviation (*x* ± *s*). Categorical variables are presented as a number and its percentage. Student's *t* test was used for continuous variables. The *P* value was set at the 0.05 level for statistical significance testing.

## Results

### Operation

In 48 patients, the rate of technique success (after the common bile duct stones were successfully removed by ERCP, the guide wire was successfully introduced into the gallbladder, and the naso-gallbladder drainage tube and/or cystic duct and duodenal papilla metal-covered stent were indwelled) was 92.86% (44/48). The four cases of failure were as follows: ① In one patient, gallbladder perforation occurred during the operation. The patient recovered well after 2 weeks of indwelling nasocystic duct drainage. No cholecystectomy has been performed thus far during the follow-up, with no clinically relevant symptoms. ② In one patient, a stone basket could not be inserted into the gallbladder after repeated attempts. ③ In one patient, the cystic duct merged into common bile duct at a low position, and the cystic duct was twisted, so the stent could not be placed. ④ In one patient, the cystic duct was extremely narrow, and the resistance to dilation along the guide wire was strong, so it was impossible for the guide wire to enter the naso-gallbladder drainage duct or to place the stent. The latter 3 patients were all transferred to surgery for laparoscopic cholecystectomy. Among the 44 patients for whom the technique was successful, clinical success was achieved in 34 patients (the technique was successful, and all gallbladder stones were removed), for a success rate of 77.27% (34/44), while the gallbladder stones of 10 patients were not completely removed. The main reasons were as follows: ① the stones in 6 patients were large, with a diameter of more than 1 cm, so they were difficult to crush with the mesh basket; ② the cystic ducts of 2 patients were thin, and the cystic duct stent was not fully expanded; and ③ sediment-like stones, found in 2 patients, were difficult to remove after repeated washing and suction.

### Symptoms and biochemical indicators

Among all patients, the abdominal pain symptoms of 47 patients were significantly relieved after the operation; the remaining 1 patient had an intraoperative gallbladder perforation, as described above. Among the other symptoms, 1 patient developed chills and fever after the operation, and the highest body temperature was 39.0°C. Two patients developed nausea and vomiting after the operation, accompanied by middle and upper abdominal pain. The postoperative liver function of all patients was statistically significantly improved compared with that before surgery (*P* < 0.05) (Table 3).

**Table 3:** Preoperative and postoperative biochemical characteristics of the patients.

Item	Preoperation	Postoperation	T value	*P* value
ALT (U/L)	219.48 ± 193.45	90.97 ± 93.28	4.1457	0.0001
AST (U/L)	129.44 ± 142.89	45.78 ± 55.19	3.7839	0.0004
TBIL (µmol/L)	45.15 ± 45.66	25.44 ± 21.81	2.6986	0.0088
GGT (U/L)	328.47 ± 329.18	219.02 ± 161.67	2.0677	0.0425
AKP (U/L)	170.36 ± 105.07	133.87 ± 67.24	2.0266	0.0461

ALT, alanine aminotransferase; AST, aspartate aminotransferase; TBIL, total bilirubin; GGT, gamma glutamyl transpeptidase; AKP, alkaline phosphatase.

### Complications

Intraoperative gallbladder perforation occurred in 1 patient, with an incidence of 2.08% (1/48). Pancreatitis occurred in 2 patients (4.17%), and acute cholangitis occurred in 1 patient (2.08%). The overall postoperative complication rate was 6.25% (3/48).

### Recurrence of cholelithiasis after GPC

Among the 34 patients who achieved clinical success, 3 were lost to follow-up (Table 3), the follow-up rate was 91.18% (31/34), and the average follow-up time was 27 months (6–40). The clinical symptoms of all patients were significantly improved after the operation. During the follow-up period, 1 patient was found to be treated with radical surgery for breast cancer, and the other patients had no new disease. A total of 5 patients (16.13%) had recurrent gallstones and no other new gallbladder disease. The recurrence rates at 12 months, 18 months, 24 months, 30 months and 36 months were 0, 3.23%, 6.45%, 12.9% and 16.13%, respectively. Four patients with recurrent cholecystolithiasis were asymptomatic, and one patient had slight right upper quadrant pain and discomfort. One of the 5 patients with recurrence underwent laparoscopic cholecystectomy when stones were found 2 years after the operation, and the remaining 4 patients were still undergoing regular review.

## Discussion

The treatment of common bile duct stones combined with cholecystolithiasis has progressed from open surgery to the current diversified treatment methods. Today, there are three main surgical methods for the treatment of common bile duct stones combined with cholecystolithiasis: open cholecystectomy + biliary tract exploration; ERCP bile duct stone removal + abdominal cavity endoscopic cholecystectomy (LC); and laparoscopic cholecystectomy + biliary exploration. For gallstones, although laparoscopic cholecystectomy is considered a safe procedure, the reported incidence of bile duct injury is still as high as 0.4%–4% (12). Moreover, as an organ of the human body, the basic role of the gallbladder is to protect the liver, gastric mucosa, gallbladder and colon from hepatotoxicity and hydrophobic bile acids and to regulate serum lipid levels (13). Approximately 5% to 40% of patients will develop postcholecystectomy syndrome, including dyspepsia, diarrhoea, bile reflux gastritis, etc. (14), and the risk of small bowel and colon tumours is slightly increased (15). These findings suggest that the preservation of a functional gallbladder has positive implications for patients. With the proposal of the expert consensus on minimally invasive endoscopic gallbladder-preserving surgery for benign gallbladder diseases (2018 edition) (5) and the promulgation of the latest edition of the guidelines for endoscopic gallbladder-preserving surgery (6), the concept of gallbladder preservation is being increasingly accepted by an increasing number of people. The purpose of gallbladder preservation and stone extraction is to maintain the integrity of this human organ, which is in line with the concept of noninvasiveness.

Since Kellett et al. (16) first proposed percutaneous gallbladder-preserving lithotripsy in 1988, it has gradually developed into laparoscopic gallbladder-preserving surgery (17), laparoscopic combined flexible choledochoscopy (18), natural orifice transluminal endoscopic surgery (NOTES) gallbladder preservation (19) and endoscopic ultrasonography-assisted gallbladder preservation (20). Gallbladder-preserving cholecystolithotomy (GPC), while being the least invasive, also provides a better choice for some specific patients, such as those who cannot tolerate cholecystectomy. However, although these techniques preserve the gallbladder and have their own advantages and indications, they all inevitably require incision of the gallbladder, and the path for stone extraction needs to penetrate the gallbladder wall. Bleeding, bile leakage and cholecystitis caused by damage to the gallbladder wall or some natural orifices may occur in transmural GPC (21, 22). It is well known that interstitial cells of Cajal (ICC) are a type of interstitial cell mainly distributed in the digestive tract. They are the pacemaker cells and signal transduction cells of the digestive tract, which stimulate and promote the peristalsis of the digestive tract. There is evidence that, in gallstone disease, the reduced number of interstitial cells of Cajal may affect the motor function of the gallbladder (23). Cajal-like cells are distributed throughout the gallbladder. Transmural GPCs require an incision on the gallbladder, and scar repair in the later stage may affect the motor function of the gallbladder to a certain extent. Motor function plays an important role in preventing crystallization and the precipitation of excess cholesterol in gallbladder bile (24).

How can we reduce damage to while also protecting the gallbladder? As early as 1984, Kozarek et al. (25) reported that endoscopic naso-gallbladder drainage (ENGBD) could be used to collect bile by ERCP. In 1991, Tamada et al. (26) reported that ENGBD was used for the treatment of acute cholecystitis. Takao Itoi et al. (27) analysed and summarized several methods of gallbladder decompression in the treatment of acute cholecystitis, including percutaneous transhepatic gallbladder drainage and percutaneous transhepatic gallbladder puncture, endoscopic nasal-gallbladder drainage and endoscopic transpapillary gallbladder stent placement, and endoscopic ultrasound-guided gallbladder puncture naso-gallbladder drainage or stent placement. It was suggested that endoscopic naso-gallbladder drainage and endoscopic transduodenal papillary gallbladder stent placement in the treatment of acute cholecystitis were more promising. Therefore, ENGBD or endoscopic transduodenal papillary gallbladder stenting is a safe and effective minimally invasive method for the management of high-risk acute cholecystitis that can relieve obstruction, provide adequate drainage, and improve acute symptoms while preserving the gallbladder.

Inspired by this, since December 2018, for patients with common bile duct stones and cholecystolithiasis who are scheduled to undergo ERCP, our team has prospectively carried out ERCP-based transcystic gallbladder-preserving cholecystolithotomy. The differences between this study and previous studies are as follows: (1) Through the duodenal papillary approach, a stent is used to establish a stone extraction channel into the gallbladder, which does not require making an incision in the gallbladder. The motor function of the gallbladder is thus improved. ② Most of the previous gallbladder-preserving surgeries were performed on patients with only gallstones. The patients selected for gallbladder preservation in this study all had common bile duct stones and had indications for ERCP. The study found that gallbladder preservation did not increase the complications of ERCP, with an incidence of pancreatitis after ERCP of 4.17%, and the incidence of cholangitis was 2.08%. The most recent literature reports that the incidence of pancreatitis after ERCP is approximately 9.7%, and it can even reach 14.7% for high-risk groups (28), and the incidence of acute cholangitis after ERCP is 0.5% to 3% (29). ③ The requirements for common bile duct conditions are as follows. First, there should be no stenosis at the lower end of the common bile duct to ensure that the common bile duct stones can be removed smoothly and to facilitate the placement of the gallbladder stent through the duodenal papilla. Second, the size of the common bile duct stones should not exceed 1.2 cm. In this way, mini-incision duodenal papillary sphincterotomy and/or small balloon dilation (diameter <12 mm) can generally preserve papillary sphincter function, and the preservation of Oddi sphincter function is of great significance for the protection of gallbladder function (30). ④ Even with acute pancreatitis, if the aetiology is biliary, gallstones can still be removed when the pancreatitis is stable. Thus, for patients with biliary pancreatitis, it is no longer necessary to be concerned about and to determine when to perform surgery for the removal of the gallbladder.

Of course, there are also some problems that were discovered in the course of this study. First, cystic duct cannulation remains the most challenging procedure. The cystic duct opening was easily identified with the help of SpyGlass peroral cholangioscopy, which subsequently increased the success rate of cystic duct cannulation and gallbladder stent placement from an initial 53% to 92.86%, but cystic duct cannulation failed in 4 patients in our study. A narrow cystic duct, a tortuous cystic duct, and a sharp cystic duct angle can all lead to failure (31). Second, a gallstone diameter greater than 1 cm was one of the main reasons for clinical failure. In the future, these patients will need an additional lithotripsy process to reduce the stone size to less than 1 cm to improve the clinical success rate of this technique.

In terms of the recurrence rate of stones, the literature reports that the stone recurrence rate of endoscopic minimally invasive cholecystolithotomy (EMIC) varies greatly from 4.92% to 40.0% (10). We followed up 34 patients with clinical success, and 5 patients (16.13%) had gallstone recurrence. The possible reasons for recurrence are as follows: (1) The sample size was small. (2) The patients did not strictly take ursodeoxycholic acid (URSO) for 6 months after the operation. URSO not only can increase the solubility of cholesterol in bile but also can improve the contractility of gallbladder smooth muscle by reducing the cholesterol content in the plasma membrane of muscle cells and biochemical parameters of oxidative stress (32), thereby reducing the recurrence rate of stones. (3) There was no restriction for study inclusion on the basis of the number of gallstones. Among the 5 patients with recurrence, 4 had more than 10 gallbladder stones indicated by preoperative B-ultrasound. Villanova N et al. (33) reported that patients with multiple stones before surgery had a higher recurrence rate after stone removal treatment.

In conclusion, ERCP-based gallbladder-preserving cholecystolithotomy (ERCP-GPC) can avoid complications related to cholecystectomy, completely preserve the structure of the gallbladder, and effectively protect gallbladder function. For patients who are suitable for gallbladder preservation, ERCP-GPC is a safe, feasible and effective new minimally invasive treatment method. Of course, to provide more evidence-based medical evidence, it is necessary to carry out prospective, controlled studies with larger samples and longer follow-up periods.

## Data Availability

The original contributions presented in the study are included in the article/Supplementary Material, further inquiries can be directed to the corresponding author.

## References

[1] Hjaltadottir K Haraldsdottir KH Moller PH. Gallstones-review. Laeknabladid. (2020) 106(10):464–72 (Icelandic). 10.17992/lbl.2020.10.602 32991309

[2] ERCP Group, Chinese Society of Digestive Endoscopology; Biliopancreatic Group, Chinese Association of Gastroenterologist and Hepatologist; National Clinical Research Center for Digestive Diseases. Chinese Guidelines for ERCP (2018). Zhonghua Nei Ke Za Zhi. (2018) 57(11):772–801 (Chinese). 10.3760/cma.j.issn.0578-1426.2018.11.002 30392234

[3] Girometti R Brondani G Cereser L Como G Del Pin M Bazzocchi M Post-cholecystectomy syndrome: spectrum of biliary findings at magnetic resonance cholangiopancreatography. Br J Radiol. (2010) 83(988):351–61. 10.1259/bjr/99865290 20335441 PMC3473449

[4] Stathopoulos P Zundt B Spelsberg FW Kolligs L Diebold J Goke B Relation of gallbladder function and Helicobacter pylori infection to gastric mucosa inflammation in patients with symptomatic cholecystolithiasis. Digestion. (2006) 73(2–3):69–74. 10.1159/000092746 16641551

[5] Gallbladder-Preserving Comittee EDBoCMDA. Expert consensus on choledochoscopic gallbladder-preserving surgery for benign gallbladder diseases (2018 edition). China J Endoscopy. (2018) 24(9):106–12 (Chinese). 10.3969/j.issn.1007-1989.2018.09.022

[6] The Gallbladder-Preserving Surgery Committee Endoscopy Specialist Branch of Chinese Medical Doctor Association. The clinical guideline for choledochoscopic gallbladder-preserving surgery (2021 edition). China J Endoscopy. (2021) 27(8):1–9 (Chinese). 10.12235/E20210460

[7] Itoi T Sofuni A Itokawa F Kurihara T Tsuchiya T Moriyasu F Preoperative diagnosis and management of thick-walled gallbladder based on bile cytology obtained by endoscopic transpapillary gallbladder drainage tube. Gastrointest Endosc. (2006) 64(4):512–9. 10.1016/j.gie.2006.01.024 16996341

[8] McCarthy ST Tujios S Fontana RJ Rahnama-Moghadam S Elmunzer BJ Kwon RS Endoscopic transpapillary gallbladder stent placement is safe and effective in high-risk patients without cirrhosis. Dig Dis Sci. (2015) 60(8):2516–22. 10.1007/s10620-014-3371-4 25287001

[9] Maekawa S Nomura R Murase T Ann Y Oeholm M Harada M. Endoscopic gallbladder stenting for acute cholecystitis: a retrospective study of 46 elderly patients aged 65 years or older. BMC Gastroenterol. (2013) 13:65. 10.1186/1471-230X-13-65 23586815 PMC3675408

[10] Hao Y Yang Z Yang H Hong J. Gallbladder-preserving cholecystolithotomy. Expert Rev Gastroenterol Hepatol. (2022) 16(3):265–72. 10.1080/17474124.2022.2047650 35236201

[11] Working Group IAP/APA Acute Pancreatitis Guidelines. IAP/APA evidence-based guidelines for the management of acute pancreatitis. Pancreatology. (2013) 13(4 Suppl 2):e1–15. 10.1016/j.pan.2013.07.063 24054878

[12] Thurley PD Dhingsa R. Laparoscopic cholecystectomy: postoperative imaging. Am J Roentgenol. (2008) 191(3):794–801. 10.2214/AJR.07.3485 18716112

[13] Turumin JL Shanturov VA Turumina HE. The role of the gallbladder in humans. Rev Gastroenterol Mex. (2013) 78(3):177–87. 10.1016/j.rgmx.2013.02.003 23683886

[14] Jaunoo SS Mohandas S Almond LM. Postcholecystectomy syndrome (PCS). Int J Surg. (2010) 8(1):15–7. 10.1016/j.ijsu.2009.10.008 19857610

[15] Goldacre MJ Wotton CJ Abisgold J Yeates DG Collins J. Association between cholecystectomy and intestinal cancer: a national record linkage study. Ann Surg. (2012) 256(6):1068–72. 10.1097/SLA.0b013e3182759efb 23154397

[16] Kellett MJ Wickham JE Russell RC. Percutaneous cholecystolithotomy. Br Med J (Clin Res Ed). (1988) 296(6620):453–5. 10.1136/bmj.296.6620.453 3126859 PMC2545040

[17] Gao DK Wei SH Li W Ren J Ma XM Gu CW Totally laparoscopic gallbladder-preserving surgery: a minimally invasive and favorable approach for cholelithiasis. Exp Ther Med. (2015) 9(2):395–8. 10.3892/etm.2014.2107 25574204 PMC4280921

[18] Tan YY Zhao G Wang D Wang JM Tang JR Ji ZL. A new strategy of minimally invasive surgery for cholecystolithiasis: calculi removal and gallbladder preservation. Dig Surg. (2013) 30(4–6):466–71. 10.1159/000357823 24481280

[19] Liu B Du B Pan Y. Video of the month: transrectal gallbladder-preserving cholecystolithotomy via pure natural orifice transluminal endoscopic surgery: first time in humans. Am J Gastroenterol. (2015) 110(12):1655. 10.1038/ajg.2015.266 26673494

[20] Ge N Wang S Wang S Wang G Liu X Guo J Endoscopic ultrasound-assisted cholecystogastrostomy by a novel fully covered metal stent for the treatment of gallbladder stones. Endosc Ultrasound. (2015) 4(2):152–5. 10.4103/2303-9027.156749 26020052 PMC4445175

[21] Ye L Liu J Tang Y Yan J Tao K Wan C Endoscopic minimal invasive cholecystolithotomy vs laparoscopic cholecystectomy in treatment of cholecystolithiasis in China: a meta-analysis. Int J Surg. (2015) 13:227–38. 10.1016/j.ijsu.2014.12.014 25527194

[22] Tsuchiya T Sofuni A Itoi T. Case of successful endoscopic ultrasonography-guided gastrojejunostomy for gastric outlet obstruction caused by gallbladder carcinoma. Dig Endosc. (2019) 31(Suppl 1):66–7. 10.1111/den.13360 30994232

[23] Tan YY Ji ZL Zhao G Jiang JR Wang D Wang JM. Decreased SCF/c-kit signaling pathway contributes to loss of interstitial cells of cajal in gallstone disease. Int J Clin Exp Med. (2014) 7(11):4099–106. eCollection 2014. 25550919 PMC4276177

[24] Housset C Chrétien Y Debray D Chignard N. Functions of the gallbladder. Compr Physiol. (2016) 6(3):1549–77. 10.1002/cphy.c150050 27347902

[25] Kozarek R A. Selective cannulation of the cystic duct at time of ERCP. J Clin Gastroenterol. (1984) 6(1):37–40. PMID: 6699392

[26] Tamada K Seki H Sato K Kano T Sugiyama S Ichiyama M Efficacy of endoscopic retrograde cholecystoendoprosthesis (ERCCE) for cholecystitis. Endoscopy. (1991) 23(01):2–3. 10.1055/s-2007-1010596 2009832

[27] Itoi T Coelho-Prabhu N Baron TH. Endoscopic gallbladder drainage for management of acute cholecystitis. Gastrointest Endosc. (2010) 71(6):1038–45. 10.1016/j.gie.2010.01.026 20438890

[28] Kochar B Akshintala VS Afghani E Elmunzer BJ Kim KJ Lennon AM Incidence, severity, and mortality of post-ERCP pancreatitis: a systematic review by using randomized, controlled trials. Gastrointest Endosc. (2015) 81(1):143–149.e9. 10.1016/j.gie.2014.06.045 25088919

[29] Wan X Chen S Zhao Q Li T Luo S Cai X The efficacy of temporary placement of nasobiliary drainage following endoscopic metal stenting to prevent post-ERCP cholangitis in patients with cholangiocarcinoma. Saudi J Gastroenterol. (2018) 24(6):348–54. 10.4103/sjg.SJG_94_18 30027911 PMC6253912

[30] Liu DQ Zhang H Xiao L Zhang BY Liu WH. Single-operator cholangioscopy for the treatment of concomitant gallbladder stones and secondary common bile duct stones. J Gastroenterol Hepatol. (2019) 34(5):929–36. 10.1111/jgh.14468 30216536

[31] Ridtitid W Piyachaturawat P Teeratorn N Angsuwatcharakon P Kongkam P Rerknimitr R. Single-operator peroral cholangioscopy cystic duct cannulation for transpapillary gallbladder stent placement in patients with acute cholecystitis at moderate to high surgical risk (with videos). Gastrointest Endosc. (2020) 92(3):634–44. 10.1016/j.gie.2020.03.3866 32330504

[32] Guarino MP Cong P Cicala M Alloni R Carotti S Behar J. Ursodeoxycholic acid improves muscle contractility and inflammation in symptomatic gallbladders with cholesterol gallstones. Gut. (2007) 56(6):815–20. 10.1136/gut.2006.109934 17185355 PMC1954869

[33] Villanova N Bazzoli F Taroni F Frabboni R Mazzella G Festi D Gallstone recurrence after successful oral bile acid treatment. A 12-year follow-up study and evaluation of long-term postdissolution treatment. Gastroenterology. (1989) 97(3):726–31. 10.1016/0016-5085(89)90644-6 2753332

